# Creation of Negatively Charged Boron Vacancies in Hexagonal Boron Nitride Crystal by Electron Irradiation and Mechanism of Inhomogeneous Broadening of Boron Vacancy-Related Spin Resonance Lines

**DOI:** 10.3390/nano11061373

**Published:** 2021-05-22

**Authors:** Fadis F. Murzakhanov, Boris V. Yavkin, Georgiy V. Mamin, Sergei B. Orlinskii, Ivan E. Mumdzhi, Irina N. Gracheva, Bulat F. Gabbasov, Alexander N. Smirnov, Valery Yu. Davydov, Victor A. Soltamov

**Affiliations:** 1 Institute of Physics, Kazan Federal University, Kremlevskaya 18, 420008 Kazan, Russia; murzakhanov.fadis@yandex.ru (F.F.M.); boris.yavkin@gmail.com (B.V.Y.); George.Mamin@kpfu.ru (G.V.M.); orlinskii@list.ru (S.B.O.); IEMumdzhi@kpfu.ru (I.E.M.); subirina@gmail.com (I.N.G.); bulgabbasov@gmail.com (B.F.G.); 2 Division of Solid State Physics, Ioffe Institute, Politekhnicheskaya 26, 194021 St. Petersburg, Russia; alex.smirnov@mail.ioffe.ru (A.N.S.); Valery.Davydov@mail.ioffe.ru (V.Y.D.)

**Keywords:** van der Waals materials, hBN, boron vacancies, optical spin polarization, electron spin resonance, crystalline quality control

## Abstract

Optically addressable high-spin states (S ≥ 1) of defects in semiconductors are the basis for the development of solid-state quantum technologies. Recently, one such defect has been found in hexagonal boron nitride (hBN) and identified as a negatively charged boron vacancy ($V_{B}^{-}$). To explore and utilize the properties of this defect, one needs to design a robust way for its creation in an hBN crystal. We investigate the possibility of creating $V_{B}^{-}$ centers in an hBN single crystal by means of irradiation with a high-energy (E = 2 MeV) electron flux. Optical excitation of the irradiated sample induces fluorescence in the near-infrared range together with the electron spin resonance (ESR) spectrum of the triplet centers with a zero-field splitting value of *D* = 3.6 GHz, manifesting an optically induced population inversion of the ground state spin sublevels. These observations are the signatures of the $V_{B}^{-}$ centers and demonstrate that electron irradiation can be reliably used to create these centers in hBN. Exploration of the $V_{B}^{-}$ spin resonance line shape allowed us to establish the source of the line broadening, which occurs due to the slight deviation in orientation of the two-dimensional B-N atomic plains being exactly parallel relative to each other. The results of the analysis of the broadening mechanism can be used for the crystalline quality control of the 2D materials, using the $V_{B}^{-}$ spin embedded in the hBN as a probe.

## 1. Introduction

Optically polarized spin states of defects in wide band gap semiconductors are one of the major building blocks of contemporary solid-state quantum technologies [1,2]. The main idea of their use is that the high spin state (S ≥ 1) of the defect, which is split in a zero magnetic field, can be initialized, manipulated, and subsequently read out by means of optical and radio frequencies, providing the possibility to control the spin. Intensive exploration of this idea began after the discovery of the unique spin-optical properties of the negatively charged nitrogen vacancy defect (NV^−^ defect) in diamond [3,4] and gave birth to several new scientific areas, such as room temperature spintronics [5], quantum sensing [6,7], and quantum information processing with defects [7,8].

Since then, the phenomenon of the optically induced spin polarisation of defects’ spin sublevels through spin-dependent recombination has triggered a plethora of studies in fundamental [8,9] and applied physics [6,10], motivating an incessant flow of theoretical [2,11,12] and experimental [13,14,15,16,17] work devoted to the identification of such defects and the establishment of their main spin and optical properties, followed by the development of protocols that should allow for the efficient use of these properties in quantum technologies [1,2]. Silicon–carbon divacancies [13,15] and negatively charged silicon vacancies in silicon carbide [14,15,16] are just a few examples. All these defects are characterized by narrow zero phonon lines that interconnect their ground and excited states, ranging from the visible up to a near-infrared spectral range; the ground state of these defects is split even in the zero magnetic field (zero-field splitting) due to the spin-spin interaction. Moderate GHz or MHz (depending on the defect type) values of the zero-field splitting (ZFS) allows for controlling the defects’ spin down to the single defect level [14,18]. A unique combination of these properties allows for the development of quantum networks, as well as quantum sensors and room temperature operated masers [16,18,19].

**Figure 1:**
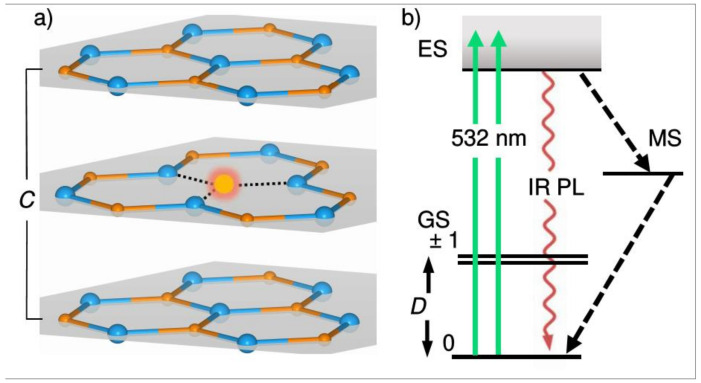
(**a**) Schematic representation of the hBN lattice. Boron vacancy is shown as the glowing spot. Nitrogen and boron are shown in blue and brown colors, respectively. The basal 2D planes (grey) of *sp*^2^-hybridized Nitrogen–Boron atoms are stacked perpendicular to the hexagonal ***c***-axis. (**b**) $V_{B}^{-}$ center energy levels diagram in the absence of external static magnetic field and the scheme of optical pumping cycle of the ground state (GS) m_s_ = 0 spin sublevel. Excitation (green) transfers the system into the excited state (ES). Radiative recombination (purple) and spin-dependent nonradiative intersystem crossings decay to the GS via metastable state (MS) (dashed lines). *D* denotes the ZFS.

Extensively studied in three-dimensional (3D) crystals characterized by *sp*^3^-hybridized atoms, such as diamond and silicon carbide, defects possessing spin-dependent optical recombination have only very recently been found in hexagonal boron nitride (hBN), which is a 2D van der Waals (vdW) material formed by the atomic planes of *sp*^2^-hybridized atoms, interconnected through van der Waals forces [20,21,22,23]. hBN is the ultra-wide (UW) bandgap (≈6 eV) material [24] which can host a large variety of atomic impurities (or point defects), which give rise to optical transitions, well below its bandgap [25]. These properties combined with the possibility to create atomically thin 2D layers due to the weakness of the interlayer coupling in vdW materials allow for the study of the properties of spin-related phenomena at the limit of the condensed state of matter. While spin-dependent optical recombination and optically detected magnetic resonance has been observed for several types of defects in hBN [20,21,22,23], so far only one defect was rigorously identified, and its microscopic structure was established by means of electron spin resonance (ESR) and optically detected magnetic resonance (ODMR) [21]. This defect is the negatively charged boron vacancy ($V_{B}^{-}$), a missing boron atom having three equivalent nitrogen atoms as the nearest neighbours, as schematically shown in Figure 1a. This defect forms a spin-triplet (S = 1) ground state (GS) which is characterized by the zero-field splitting value *D* = 3.5 GHz [21,26,27]. Optical excitation with green light induces a pronounced near-infrared photoluminescence (IR PL) and leads to a predominant population of the m_s_ = 0 spin sublevel through the spin-dependent recombination pathway via the metastable state (MS) in its excitation-recombination cycle. All the properties mentioned above are summarized in Figure 1b. Together with pronounced spin coherence properties [28], this defect is currently considered to be a very promising candidate for quantum technologies.

To explore and utilize the optical and spin properties of $V_{B}^{-}$ defects, methods for their reliable creation should be developed. To date, several types of irradiation techniques were used for the creation of $V_{B}^{-}$ centers in hBN lattice. They are neutron irradiation [21,29], irradiation with a focused beam of different ions [21,30], and irradiation with the femtosecond laser pulses [31]. In this article, we explore the possibility of creating these defects by means of high-energy electron irradiation. This type of irradiation technique makes it possible to avoid clustering of the defects, and it is widely used for the creation of a highly homogeneous distribution of the point defects in solids. Utilizing microphotoluminescence (µPL) and electron spin resonance (ESR) techniques, we found electron irradiation to be a robust way to create the boron vacancies in the negative charge state. 

After establishing a reliable means of creating $V_{B}^{-}$ centers in hBN, we focused on the exploration of the spin resonance line broadening mechanism associated with this center. The latter is important since the broadening of the spin resonance transitions serves as the limitation of the spin-coherence time (T_2_) [32]—one of the central properties of any quantum system that possesses an intrinsic magnetic moment [1,2]. This has been demonstrated for the NV^−^ centers in diamonds and negatively charged silicon vacancies ($V_{\mathrm{Si}}^{-}$) in SiC. While NV^−^ ESR transitions are broadened due to the fluctuations of the local magnetic fields [33], the transitions of $V_{\mathrm{Si}}^{-}$ centers are broadened due to the variation of the zero-field splitting around the mean value [34]. The broadening mechanism of the $V_{B}^{-}$ resonance transitions established in the current work differs from the two mechanisms mentioned above, and we established that it arises because the weak interlayer vdW bonding force is not sufficient to protect the 2D lattice planes from slight misalignment due to being perpendicular to the hexagonal ***c***-axis. In other words, the broadening is induced by the deviation of the relative orientation of the 2D nitrogen–boron planes from parallel orientation. It should be noted that the observed broadening is completely determined by the crystalline quality of hBN and the inherent two-dimensional structure of this material: that is, the spin resonance linewidth of any defect (or impurity) with a spin state greater than S = 1/2 will undergo a broadening of such type. Additionally, the analysis of the broadening reveals important peculiarities of the hBN crystalline perfection. Thus, the high spin state of defects embedded in hBN crystal can serve as a tool to control the hBN crystalline quality, with further applications for 2D heterostructures utilizing hBN as the encapsulation material.

## 2. Materials and Methods

The hexagonal boron nitride single crystals with dimensions of 1 mm × 1 mm × 0.15 mm used in this study were commercially produced by the HQ Graphene company. The samples were irradiated at room temperature with 2 MeV electrons to a total dose 6 × 10^18^ cm^−2^. No annealing treatments were applied to the irradiated samples. One sample was not subjected to irradiation and was used as the reference sample. The optical properties of the samples were studied at room temperature by microphotoluminescence (µ-PL) and Raman measurements using a T64000 (Horiba Jobin-Yvon, Lille, France) spectrometer equipped with a confocal microscope and a silicon CCD cooled by liquid nitrogen. The line at λ = 532 nm (2.33 eV) of a Nd:YAG laser (Torus, Laser Quantum, Inc.; Edinburg, UK) was used as the excitation source. Micro-Raman studies were carried out at the same sample point as the µ-PL ones, and backscattering geometry was used in both measurements. The laser beam along the normal to the surface was focused by a Leica PL FLUOTAR 50× objective lens (NA = 0.55) into a spot size of ~2 µm in diameter, with a power density of 10 kW/cm^−2^ on the sample. We used a 600 lines/mm grating for the µ-PL and an 1800 lines/mm grating for the Raman measurements. In a low frequency region of the Raman spectrum, the Rayleigh line was suppressed using three BragGrate notch filters (OptiGrate Corp.; Oviedo, FL, USA) with an OD = 4 and a spectral bandwidth <0.3 nm. Electron spin resonance (ESR) studies of the sample were made at X-band frequencies (≈9.4 GHz) on a commercial Bruker ESP300 (Bruker Biospin, Billerica, MA, USA) spectrometer equipped with an Oxford Instruments ESR-9 (Abingdon, UK) liquid helium flow cryostat. The simulation of the measured ESR data was performed using EasySpin (5.2.29) software [35].

## 3. Results

We start with the characterization of the material by means of Raman spectroscopy. The hBN symmetry is described by the space group P6_3_/mmc (#194) (point group = D_6h_). There are two formula units per primitive cell. Boron and nitrogen atoms occupy the 2c and 2d positions, respectively. Boron and nitrogen atoms induce the following mechanical representation: Γ = Γ_ac_ + Γ_opt_ = 2(*A*_2*u*_ + *B*_1*g*_ + *E*_2*g*_ + *E*_1*u*_), where Γ_ac_ = *A*_2*u*_ + *E*_1*u*_ and Γ_opt_ = *A*_2*u*_ + *2B*_1*g*_ + 2*E*_2*g*_ + *E*_1*u*_. Consequently, there are six optical modes: *A_2u_* + *E_1u_* are infrared-active optical modes, 2*B*_1*g*_ modes are silent optical modes, and 2*E*_2*g*_ are Raman-active optical modes [36].

Thus, a Raman spectrum of hBN should contain two doubly degenerate E_2g_ phonon modes. In a high-energy $E_{2g}^{\left(2\right)}$ symmetry Raman phonon, boron and nitrogen atoms in each plane move in opposite directions. In a low-energy $E_{2g}^{\left(1\right)}$ symmetry Raman phonon, two B-N planes move relative to each other.

**Figure 2:**
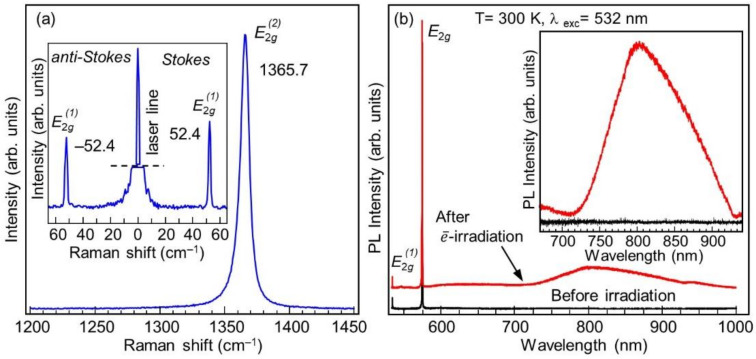
(**a**) Raman spectrum of electron-irradiated hBN in the range of $E_{2g}^{\left(2\right)}$ symmetry phonon. The inset shows the low-frequency spectra for the Stokes and anti-Stokes $E_{2g}^{\left(1\right)}$ symmetry phonon. (**b**) Room temperature µ-PL spectra measured under λ = 532 nm excitation for the reference hBN (black) and for the electron-irradiated one (red). Inset shows an enlarged scale PL band around λ = 810 nm in the enlarged scale.

Since the Raman spectra of the initial and electron-irradiated samples have very similar characteristics, Figure 2a shows only the Raman spectrum of the irradiated sample. A single peak at a frequency of 1365.7 cm^−1^ with a full width at half maximum (FWHM) of about 8 cm^−1^ originates from the in-plane $E_{2g}^{\left(2\right)}$ symmetry phonon mode of hBN. The insert to Figure 2a shows two ultra-low-frequency lines at −52.4 cm^−1^ (anti-Stokes) and 52.4 cm^−1^ (Stokes) with a FWHM of about 1.3 cm^−1^, which originate from the interlayer shear $E_{2g}^{\left(1\right)}$ symmetry phonon mode of hBN. No other lines were observed in the 5–2000 cm^−1^ range for the reference nor for the electron irradiated hBN samples. It may be concluded that the Raman spectra presented in Figure 2a are typical for a crystalline hBN without any inclusions [37]. It must be noted that the state-of-the-art crystalline quality of hBN differs significantly depending on the manufacturer. The best crystalline perfection is expected for crystals produced by the National Institute of Materials Science (NIMS, Tsukuba, Japan) (FWHM of Raman $E_{2g}^{\left(2\right)}$ High-frequency line is 7.1 cm^−1^); the perfection of single crystals produced by HQ Graphene is lower but is still of a high grade (FWHM of $E_{2g}^{\left(2\right)}$ High-frequency line is 8.2 cm^−1^) [37].

The high-energy optical response of the sample was modified significantly by electron irradiation, as can be seen in Figure 2b. A near-infrared wide PL band around λ = 810 nm is observed in the sample after the irradiation procedure previously shown to arise due to the presence of $V_{B}^{-}$ defects [21]. We also note that the 532 nm (2.33 eV) excitation used for the excitation of the PL spectrum is within the optical absorption band of $V_{B}^{-}$ defects, as was previously revealed by low-temperature photoluminescence excitation spectroscopy [30]. There, it was also shown that the PL spectrum of $V_{B}^{-}$ defects at a low temperature does not possess any narrow peaks within the emission wavelength. This PL band is the first signature of the negatively charged boron vacancy defects in the hBN crystal. After electron irradiation, a weak wide PL band in the visible (VIS) spectral range appears. However, in previously published papers devoted to the PL properties of the $V_{B}^{-}$ defect, this broad band was not observed [21,30]. In Ref. [31] it was shown that PL features in the VIS range can be observed together with the 810 nm band, although without any correlations with the latter. The origin of this VIS PL band is beyond the scope of this work.

**Figure 3:**
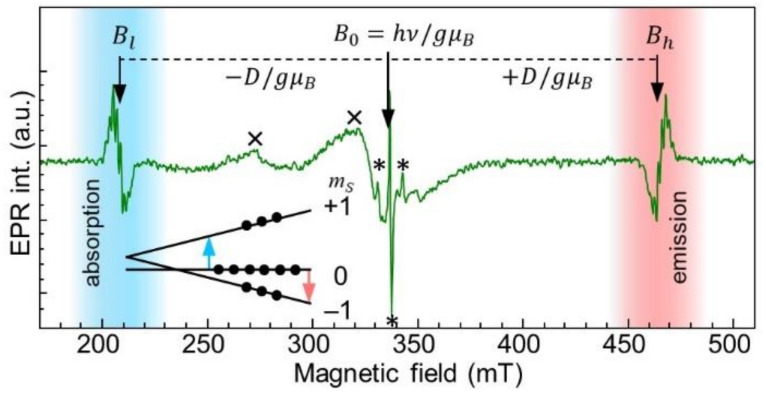
X-band ESR spectrum registered in the electron-irradiated hBN sample at T = 35 K under λ = 532 nm excitation at ***B*** || ***c***, azimuthal angle θ = 0°. Triplet fine-structure lines together with the magnetic field corresponding to the center of gravity are labelled as $B_{l}$, $B_{h}$, and $B_{0}$, respectively. Blue- and red-shaded areas indicate absorption and emission of the MW power, respectively. Inset shows *S* = 1. Zeeman levels diagram on which optically induced predominant population of the m_s_ = 0 in the triplet ground state is shown. Allowed magnetic dipole transitions are indicated by arrows. With symbol **×**, artifacts arising from the resonator are marked. EPR signals corresponding to the unidentified paramagnetic centers in hBN are labeled with asterisks.

We then focused on the electron spin resonance studies of the irradiated sample. Figure 3 shows the ESR spectrum measured under 532 nm excitation in the orientation of static magnetic field parallel to the hexagonal ***c***-axis (***B*** || ***c***, azimuthal angle θ = 0°). The allowed magnetic dipole transitions giving rise to the doublet of ESR lines $B_{l}$ and $B_{h}$ with the splitting in the magnetic field $\Delta B=2D/g\mu_{B}=256.5\;mT$ were located symmetrically relative to the triplet spectrum center of gravity ($B_{0})$. The spectrum can be described by the axially symmetric spin-Hamiltonian given in Equation (1) with the following parameters: S = 1, g = 2.0008, D = 3.6 GHz, E = 50 MHz:

(1) H=gμBB·S+DSz2−23+ESx2−Sy2

where S is the electron spin operator with S = 1 being the total spin of $V_{B}^{-}$; $\mu_{B}$ is the Bohr magneton; B is the static magnetic field; D and E describe the zero-field splitting interaction; $S_{z},$ $S_{x}$ and $S_{y}$ the total spin-triplet operators; and the *z*-axis is collinear with the hexagonal ***c***-axis. 

The values of g, D, and E are in good agreement with those previously established for the negatively charged boron vacancy in the hBN crystal. It should be noted that following previous studies [21,38,39], ZFS of the $V_{B}^{-}$ center is temperature dependent, exhibiting pronounced *D*(T) behavior. The *D* = 3.6 GHz measured in our experimental conditions (T = 35 K) is in line with the $V_{B}^{-}$ *D*(T) dependence established in the abovementioned works. As has been previously pointed out, under optical excitation a predominant population of the m_s_ = 0 spin sublevel in the $V_{B}^{-}$ GS can be created, due to the spin-dependent recombination from the ES into the GS through metastable state. This optically induced deviation of the ground-state spin sublevel population from Boltzmann statistics manifests itself in the phase reversal character of the ESR fine-structure lines highlighted with blue and red areas in Figure 3. The emission of microwave power rather than absorption is observed for the m_s_ = 0→m_s_ = −1 transition. These observations are signatures of optically addressable spin states of the $V_{B}^{-}$ defect present in the hBN crystal, demonstrating the electron irradiation reliability for the creation of $V_{B}^{-}$ defects in hBN.

**Figure 4:**
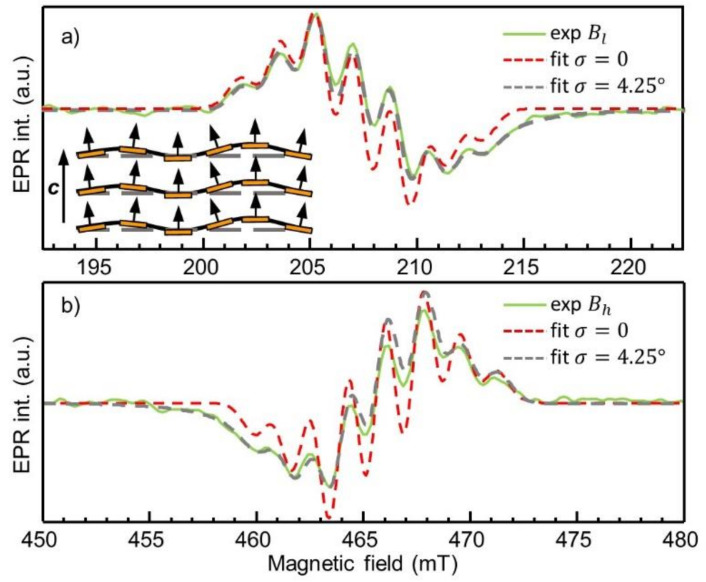
The fine-structure lines $B_{l}$ (**a**) and $B_{h}$ (**b**) from Figure 3 are shown in the enlarged scale with solid green-colored lines. Red dashed lines show the simulation of the $B_{l}$ and $B_{h}$ when only *A* = 47 MHz HF interaction constant with three equivalent ^14^N atoms is considered. The same simulation is shown by a grey color, but taking into account broadening mechanism through the standard deviation σ of the hBN layers from the plane perpendicular to the ***c***-axis by σ = 4.25°. Inset in (**a**) is a sketch of hBN layers misalignment in angle relative to the ***c***-axis. Grey dashed lines show the perfect alignment of 2D layers. The brownish rectangles provide an overview of the deviation of basal planes from perpendicular orientation to the ***c***-axis.

Components of the ESR spectrum shown in Figure 4a,b demonstrate the seven-line structure induced by the hyperfine interaction of the $V_{B}^{-}$ electron spin with three equivalent ^14^N nitrogen nuclei (^14^N nuclear spin is *I* = 1) nearest to the vacancy. The number (*N*) of lines is in correspondence with the number (n) of equivalent nuclei and given by *N* = 2n*I* + 1, where *I* = 1. The constant *A* characterizing this interaction is equal to 47 MHz, established by fitting the spectra using spin-Hamiltonian of Equation (1), which includes an additional $\sum_{k}A_{k}SI_{k}$ term. This value is in agreement with that previously measured in Ref. [21] and confirmed by DFT calculations [25,26]. The best fits are shown with a red-dotted envelope of the ESR spectra for both the m_s_ = 0→m_s_ = −1 and m_s_ = 0→m_s_ = +1 fine stricture transitions. 

Attention must be drawn to the experimentally observed line shapes of the $B_{l}$ and $B_{h}$ shown in Figure 4a,b in the enlarged scale. These lines are stretched along the field axis towards the triplet spectrum center of gravity. This observation cannot be described only by taking into account the 47 MHz hyperfine interaction, as can be seen from corresponding simulations presented in Figure 4a,b. Namely, while the constant *A* = 47 MHz describes positions of the hyperfine-structure lines reasonably well, the relative intensities of the hyperfine lines together with the asymmetry of the fine-structure ESR lines are not satisfactorily reproduced. Broadening mechanisms, such as unresolved ligand hyperfine interactions [32,40] and the presence of local strain (or local magnetic) fields inducing a significant deviation in g-factors [41] and *D* values [32,42] from their ideal (theoretical/canonical) values, are not appropriate to explain the observed line shape, since all these mechanisms do not lead to the asymmetry of the ESR line.

The concentration of $V_{B}^{-}$ defects, expressed as the number of spins/cm^3^, in the electron-irradiated hBN sample was determined by comparison with a standard (reference) sample with known concentration of paramagnetic centers (Coal with C*_Coal_* = 8.3 × 10^18^ spins/cm^3^ of free radicals S = 1/2 and g ≈ 2.003). The EPR signal intensities of the free radicals in the reference sample and of $V_{B}^{-}$ centers in the hBN sample were determined by the integration of the absorption spectra over their spectral ranges. A comparison of the integral intensities of the two EPR signals obtained under the same experimental conditions provides information about the concentration of $V_{B}^{-}$ centers. In our case, the integral intensities differ from each other by about 13.8 times, i.e., $\frac{I_{Coal}}{I_{V_{B}^{-}}}=13.8$. Defined in such a way, $V_{B}^{-}$ concentration in the hBN sample is 6 × 10^17^ spins/cm^3^. We note that EPR signatures of defects at the nitrogen site previously reported in Refs. [43,44,45] are not seen in the EPR spectrum of Figure 4. However, we cannot exclude the lines marked with asterisks to be a type of nitrogen-site-related paramagnetic center, though their detailed study is beyond the scope of this work.

## 4. Discussion

The broadening mechanism can be anticipated from the character of the asymmetry it induced for each $B_{l}$ and $B_{h}$ line—that is, the abovementioned stretching towards the ESR triplet spectrum center of gravity can be qualitatively explained if we assume a slight deviation in 2D plain orientation from exactly perpendicular to the ***c***-axis (see the inset in Figure 4a). In such a case, the static magnetic field applied collinear with the nominal ***c***-axis for the major number of hBN layers, while some of the layers were characterized by their own local direction of the hexagonal axis. Thus, the angle theta *θ* becomes averaged between all possible misalignment angles within a 3σ, inducing the observed type of the broadening. Indeed, the resonant magnetic fields $B_{l}$ and $B_{h}$ for the axial triplet center follow the angular dependence $B\left(\theta \right)=\frac{1}{g\mu_{B}}\left(h\nu \mp \frac{D}{2}\left(3cos^{2}\theta -1\right)\right)$. That is, the $B_{l}$ and $B_{h}$ with the largest magnitude of the splitting ΔB are expected at the angle *θ* = 0, corresponding to the case where the major number of hBN layers are oriented perpendicular to the c axis (or to the direction of the static magnetic field ***B***). While the upper limit of the *D* value is reached (*D* = 3.6 GHz at *θ* = 0), each misalignment angle induces a shift of the resonant magnetic field only towards the center of gravity $B_{0}=\frac{h\nu }{g\mu_{B}}$ in accordance with the angular dependence. Rigorous simulation of the ESR line through the broadening mechanism described above can be implemented by taking into account the normal distribution of the angles θ: $f\left(\theta |\mu ,\sigma \right)=\frac{1}{\sigma \sqrt{2\pi }}\exp\left(\frac{-{\left(\theta -\mu \right)}^{2}}{2\sigma^{2}}\right)$. The obtained standard deviation values σ allow us to provide the discussed asymmetric broadening of the fine-structure components. From Figure 4a,b, it is seen that the simulation of the ESR line shapes, considering the broadening mechanism, matches well with the experiment and allows us to establish the standard deviation (σ) of hBN layers from the plane perpendicular to the ***c***-axis by σ = 4.25°. Quantitative analysis using the least-squares approximation approach (minimization of the sum of squared residuals of differences between an experimental data and the fitted value) allows for obtaining the most satisfactory fitting model. Through the least squares, we can quantitatively estimate the validity of the simulation with a certain value of σ in the function of the normal distribution of angles. If we consider the absence of such layer deviations (σ = 0°), the sum of squared residuals $\sum {\left(I_{exp}-y_{fit}\left(g,A,D,\sigma \right)\right)}^{2}$ is equal to 6.9 ± 0.2 (low field) and 7.1 ± 0.2 (high field). The approximation with the non-zero standard deviation (σ = 4.25°) gives significantly smaller values: 0.25 ± 0.03 and 1.01 ± 0.1 for the $B_{l}$ and $B_{h}$ lines, respectively. This confirms the proposed assumption regarding the broadening mechanism.

Owing to the large *D* value (3.6 GHz) and small individual widths of the ESR lines due to the dipole–dipole interaction (ΔB_PP_ ≈ 38 MHz), the slightest deviations of hBN layers from parallel orientation to each other can be unequivocally determined through the appearance of the asymmetrical fine-structure components broadening. The performed analysis of such a broadening mechanism provides the possibility of a practical application for the quite precise quality control of the 2D materials, using the $V_{B}^{-}$ spin embedded in hBN as a probe. Currently, there is a successful method for the precise control of the twist angle of large scale MoS_2_ homostructures. It is especially important because the interlayer twist angle of MoS_2_ films has a strong influence on both the spectroscopic properties and electronic mobility, and any deviations lead to the decrease of effects [46]. In this work, the description of the ESR triplet spectrum by involving the aforementioned broadening mechanism allows accurate determination of the spin-Hamiltonian parameters (g, *A*, and *D*) that unambiguously confirms the presence of the boron vacancy $V_{B}^{-}$ in hBN. Second, today more and more attention is paid to interphase engineering with designed deformable 2D heterostructures (i.e., highly deformable transistors including the hBN dielectric, the MoS_2_ active layer and the graphene gate) with specific properties [47], where a similar monitoring of the plane mismatching using ESR spectroscopy would be appropriate. 

## 5. Conclusions

To summarize, in the present work we have investigated the possibility of creating defects possessing optically addressable spin states in hexagonal boron nitride by means of electron irradiation. We have shown that such high-energy (*E* = 2 MeV) exposure can be used for the creation of negatively charged boron vacancies in the hBN host. The measured near-infrared photoluminescence band at λ_max_ = 810 nm and the main spin-Hamiltonian parameters from ESR spectroscopy data, including g-factor, zero-field splitting *D*, and hyperfine interaction *A* values with a distinctive manifestation of the fine-structure components due to the presence of three equivalent ^14^N nuclei, unambiguously correspond to the boron vacancy in the $V_{B}^{-}$ triplet center. The observed ESR signatures of the $V_{B}^{-}$ centers reveal the presence of the nontrivial mechanism of broadening. We analyzed the cause of this broadening in different ways and came to the conclusion that the model, which associates the broadening with a small deviation of the orientation of the two-dimensional atomic planes B-N from exactly parallel relative to each other, allows us to describe the observations of asymmetric fine-structure lines. This peculiarity of the crystal structure revealed by means of ESR spectroscopy seems to be hidden from such a sensitive analytical tool as Raman spectroscopy. This type of broadening is important when materials with interlayer bonding through van der Waals forces are studied, since the crystalline perfection of such materials can deteriorate as a result of the layers’ disorientation relative to each other. The presented quantitative analysis of the crystalline perfection using the sensitive spectroscopic parameter *D* to detect layer angular misalignments provides an opportunity to control the quality of 2D materials. The latter is important for electronic devices based on 2D heterostructures.

## Data Availability

Data is available upon request from the authors.
